# Emotion Regulation and Psychological Capital of Chinese University Students during the COVID-19 Pandemic: The Serial Mediation Effect of Learning Satisfaction and Learning Engagement

**DOI:** 10.3390/ijerph192013661

**Published:** 2022-10-21

**Authors:** Yuxi Tang, Weiguang He

**Affiliations:** College of Social Sciences, Shenzhen University, Shenzhen 518060, China

**Keywords:** COVID-19 pandemic, emotion regulation, psychological capital, learning satisfaction, learning engagement

## Abstract

The mediating mechanism between the emotion regulation and psychological capital of university students is currently unclear. This study analyzed the serial mediation of learning satisfaction and learning engagement on the relationship between the emotion regulation and psychological capital of university students during the coronavirus disease 2019 (COVID-19) pandemic. A total of 328 undergraduates and postgraduates from universities in different regions of China were surveyed through an online questionnaire. The tools used in the study were the emotion regulation questionnaire, university student learning satisfaction questionnaire, learning engagement questionnaire, and psychological capital questionnaire. The analysis revealed both direct and indirect mediation effects. It was found that emotion regulation can positively predict psychological capital. Further, learning satisfaction and learning engagement can act as mediating variables between emotion regulation and psychological capital, respectively. Learning satisfaction and learning engagement can also have a serial mediation effect between emotion regulation and psychological capital. The results show that learning support should be strengthened to improve the learning satisfaction and learning engagement of students and, consequently, enhance their psychological capital.

## 1. Introduction

The coronavirus disease 2019 (COVID-19) pandemic has profoundly impacted the physical and mental health of people, and university students have borne the brunt as well [1]. As the future is full of uncertainty and the normal academic life of students has been significantly disrupted, psychological conditions, such as the excessive anxiety, worry, and even fear experienced by university students, have become more pronounced worldwide [2]. Many countries have tried to help university students go back to their normal academic life and improve their psychological conditions to a certain extent through support methods such as remote teaching [3], mobile phone software services [4], and psychological counseling services [5]. However, there are significant differences in the economic, medical, and educational resources among different countries, and some interventions such as distance education are difficult to implement in low-income countries [6]. There is an urgent need for more effective ways to help university students improve their psychological well-being. Looking for mechanisms to help university students more effectively and conducting precise interventions may play a significant role.

During the COVID-19 pandemic, psychological capital is considered an important protective factor for mental health and can relieve anxiety and stress in people [7,8]. Previous studies have also found that psychological capital helps people cope with challenges and difficulties during the COVID-19 pandemic [9,10]. Psychological capital is defined as the level of the positive psychology of an individual [11,12], including the dimensions of hope, optimism, efficiency, and resilience [13], which can promote good mood, appropriate behavior, and work performance and is closely related to mental health [14]. In the long run, psychological capital is of great significance to the mental health and future work achievements of university students [15]. It is an important resource for self-development and can be generated through interventions. In previous studies, short-term training has been used to promote psychological capital [16,17,18], and the effectiveness of such interventions was confirmed. Moreover, some studies have called for strengthening the development of the psychological capital of students during the COVID-19 pandemic [19,20].

Emotion regulation means that people exert effect on their emotional state and react according to their goals [21,22]. It is important for individuals to reduce negative emotions and enhance positive emotions. Emotion regulation can change the disadvantageous factors caused by negative emotions and increase the experience of positive emotions. Emotion regulation may play a certain role in promoting psychological capital [23,24] and may prevent college students from suffering due to the pandemic [25]. Additionally, emotion regulation ability can affect the satisfaction of students [26,27]. Previous research has also found a correlation between emotion regulation and engagement in work situations [28,29]. There are also several studies that point to a correlation between emotion regulation and student learning engagement in education situations [30,31,32]. However, in an academic context, the mediating mechanism between emotion regulation and psychological capital is still unclear, which increases the need for further exploration. 

Learning satisfaction is the overall experience, emotion, and attitude of students towards learning [33,34]. Emotion regulation may affect job satisfaction; people who can regulate their emotions better often experience higher job satisfaction [35]. This relationship may also exist in academic situations. Previous research has found a correlation between learning satisfaction and psychological capital [36]. This implies that there may be a correlation between emotion regulation, learning satisfaction, and psychological capital. 

Learning engagement refers to the ability of students to consciously work hard, actively participate in learning, and be enthusiastic about learning [37,38]. Some studies have found a positive correlation between the emotion regulation and learning engagement of students [39,40,41]. Learning engagement is also closely related to other factors that can reflect the learning situation, such as improving the satisfaction of students [42,43]. This means that there may be a correlation between emotion regulation, learning engagement, and psychological capital.

The conservation of resources theory reflects the aversion of people to resource loss and their tendency to pursue resources [44]. Resources generally refer to various aspects required for human survival or development, including not only things that are valuable to people but also the means of obtaining them [45]. University students invest resources such as time and money into psychological growth and future employment opportunities. When university students study hard but fail to achieve the expected outcomes, it results in them losing resources and experiencing pressure [46]. Conversely, when university students are content with their learning outcomes, they are satisfied with the resources they can obtain from the existing learning activities and are motivated to enhance their learning engagement and obtain more resources. With continuous hard work, the participation of university students in learning may promote psychological capital [47]. 

According to the personal resource model, personal resources include optimism, self-esteem, and self-efficacy [48], which serve as the basis for university students to cope with challenges and pressures [49]. These types of personal resources are closely related to psychological capital [50]. Individuals with high emotion regulation may maintain an optimistic attitude and create more personal resources. Furthermore, the emotion regulation ability of university students may make them more satisfied with their learning, and consequently, they may immerse themselves more in their studies. Additionally, the learning satisfaction of university students may influence their self-efficacy [51] and optimism [52]. When university students are satisfied with their own learning conditions and invest in learning, they have the opportunity to obtain more personal resources. 

From the personal resource theory perspective, emotion regulation can promote positive emotions and a positive mentality among individuals and, consequently, cause psychological capital to accumulate. However, the mediating mechanism between the emotion regulation and psychological capital of university students is currently unclear. The objective of this study was to explore the relationship between the learning satisfaction, learning engagement, emotion regulation and psychological capital of university students and the mechanism between emotion regulation and psychological capital during the COVID-19 pandemic. We will explore the following research questions: What is the relationship between emotion regulation and psychological capital among university students during the COVID-19 pandemic? Do the learning satisfaction and learning engagement of university students mediate this relationship? Do learning satisfaction and learning engagement together play a serial mediating role in this relationship?

We propose the following four assumptions:

**Hypothesis** **1.** *There is a positive correlation between the emotion regulation ability and psychological capital of university students during the COVID-19 pandemic.*

**Hypothesis** **2.** *The learning satisfaction of university students plays a mediating role between emotion regulation and psychological capital during the COVID-19 pandemic.*

**Hypothesis** **3.** *The learning engagement of university students mediates the relationship between emotion regulation and psychological capital during the COVID-19 pandemic.*

**Hypothesis** **4.** *The learning satisfaction and learning engagement of university students constitute a series of mediation effects between emotion regulation and psychological capital during the COVID-19 pandemic.*

## 2. Materials and Methods

### 2.1. Participants and Research Process

This study recruited undergraduates and postgraduates from 29 provinces of China to participate in the study through a reliable online questionnaire platform in August 2022, which used convenience sampling method. Ethical approval was obtained in advance from the institution of the investigator. Participants were able to anonymously complete the online survey through mobile phones or web pages. All participants were informed of the reasons for the study, how the study data would be used, and any risks associated with the study before participating in the study. Informed consent for the study was provided on the first page of the questionnaire, and participants were informed that the study would only continue after they agreed to participate. 

Participants were only allowed to fill out the questionnaire once. Finally, 330 questionnaires which included four measurement scales of emotion regulation, psychological capital, learning satisfaction, and learning engagement were collected, and the average response time was about 13 min. After a review of the collected questionnaires, those with incomplete content, incomplete demographic data, or an excessively short completion time (less than 180 s) were excluded from the study. (One participant did not report their learning level, and one participant did not provide demographic data). After exclusion, 328 valid questionnaires were obtained. A sample exceeding 300 is generally considered to have a good statistical effect [53], and this study meets this requirement.

### 2.2. Measurement

The emotion regulation of university students was measured using the emotion regulation questionnaire developed by Gross and John [54], which consists of two subscales: cognitive reappraisal and inhibitory expression. There are 10 items in the questionnaire, which are scored on a 7-point Likert scale. Sample items include “I keep my emotions to myself”. A score of 1 represents very nonconforming, and a score of 7 represents very conforming. A higher total score means that the participant is more inclined to use emotion regulation strategies. The validity of the Chinese version of the scale has been confirmed, with Cronbach’s α of 0.77 to 0.85 [55]. In this study, the Cronbach’s α of the cognitive reappraisal, inhibitory expression scale, and overall scale was 0.829, 0.806, and 0.699, respectively. This scale has reported good construct and convergent validity in a large number of previous studies [56,57,58]. This same good measurement property was found in this study; for example, from the perspective of discriminant validity, the Heterotrait–Monotrait (HTMT) of correlations between the two dimensions was −0.058, which is below the recommended criterion value of 0.90 [59].

The Chinese version of the psychological capital questionnaire by Zhang et al. [60] was used in this study, which is a revised version of the positive psychological capital questionnaire developed by Luthans et al. [13]. The validity of this questionnaire has been verified in China [61,62]. The questionnaire includes five items requiring reverse scoring and 21 items requiring forward scoring including items such as “I am confident in my abilities”. Each item is scored on a 7-point Likert scale. The Cronbach’s α of this questionnaire was 0.911. The values of HTMT between the four dimensions of this scale in the study were 0.899, 0.744, 0.582, 0.754, 0.712, and 0.761, respectively, all of which were below the recommended threshold of 0.90, supporting the conclusion of good construct validity found in a large number of previous studies [63,64,65,66].

The learning satisfaction of university students was measured by the university student learning satisfaction questionnaire prepared by Li et al. [67]. The questionnaire comprises three dimensions: academic satisfaction, teaching satisfaction, and school education resources satisfaction. The validity and reliability of the Chinese version of the scale have been confirmed [68].The questionnaire contains 12 items, and each item is scored on a 5-point Likert scale, including items such as “I think what I have learned will be useful”. A score of 1 represents very noncompliant, and a score of 5 represents very compliant. The questionnaire includes one reverse scoring question and 11 forward scoring questions. The Cronbach’s α of the overall scale was 0.871. In this study, the values of HTMT between the three dimensions of this scale were 0.833, 0.892, and 0.814, respectively, which were below the recommended threshold of 0.90.

Student learning engagement is seen as a concept with a multidimensional structure [69,70,71]. Some of the scale dimensions developed by prior research have varied in structure based on diverse understandings of the concept of student learning engagement. For example, some studies have found that student engagement scales containing four dimensions of cognitive, affective, behavioral, and agentic dimensions are effective in countries such as Portugal and Iran [72,73]. However, due to the difference in social contexts and cultural traditions, we gave preference to scales that had been tested in previous studies and were widely used. The student version of the Utrecht Work Engagement Scale is considered to be one of the most widely used scales of learning engagement [74]. Therefore, the learning engagement questionnaire prepared by Schaufeli et al. [75] was used to measure the learning engagement of university students in this study. In the survey, we used the Chinese version of the scale. The Chinese version of the questionnaire was proved to be reliable, with a Cronbach’s α of 0.84 [76]. The questionnaire contains 17 items, such as “I am enthusiastic about my studies” and is scored on a 5-point Likert scale. In the present study, the Cronbach’s α of the questionnaire in this study was 0.907. Meanwhile, the values of HTMT between the three dimensions were 0.781, 0.850, and 0.802, respectively, all of which were below the suggested threshold of 0.90, indicating a high degree of discriminant validity and further supporting previous studies. Previous studies in several countries, including China, have conducted validated factor analyses of the scale and found the three-factor structure of vitality, dedication, and focus to be reliable [77,78]. As the structural validity of the scale has been validated by previous studies and for the sake of focusing on the research questions we needed, possible potential subdimensions of the learning engagement scale will not be analyzed here. 

The HTMT values of emotion regulation, psychological capital, learning satisfaction, and learning engagement were all lower than 0.90, reflecting that the measurement model has good discriminant validity (Table 1). The detailed items of the four measurement questionnaires mentioned above are presented in the Supplementary Materials.

### 2.3. Statistical Analysis

First, the average value and standard deviation of the emotion regulation, learning satisfaction, learning engagement, and psychological capital of university students were calculated. When analyzing the correlation of different variables, Pearson’s correlation coefficient was used to explore the correlation coefficient of the different variables. For the regression analysis, SPSS 27 software (IBM, Armonk, NY, USA) and Model 6 in PROCESS plug-in version 4.5 [79] were used for processing. When testing the mediation effect, the bootstrap test was mainly used to repeatedly sample 5000 times. If zero was not included in the lower bound to the upper bound of the 95% confidence interval (CI), the mediation effect was considered to be significant. In addition, the total effect, direct effect, and intermediate effect were calculated.

## 3. Results

Among the 328 participants, 262 were female university students (79.88%), and 66 were male university students (20.12%). The age range of participants was 18 to 37 years. There were 284 undergraduates, 41 master’s students, and 3 doctoral students. Participants were from 163 cities in 29 provinces in four regions of China. Participants attended 232 universities and were from 148 different professional fields. Table 2 shows the characteristics of the participants.

The average, standard deviation, and correlation of the total scores of all participants in the four variables of emotion regulation, psychological capital, learning satisfaction, and learning engagement are shown in Table 3. We found that emotion regulation is correlated with psychological capital (r = 0.395, *p* < 0.01) and learning satisfaction (r = 0.268, *p* < 0.01), learning satisfaction is correlated with learning engagement (r = 0.548, *p* < 0.01), and learning engagement is correlated with psychological capital (r = 0.684, *p* < 0.01; Table 3).

To determine whether the survey sample size could support the hypothesis test, the Monte Carlo simulation method and application provided by Schoemann and A. M et al. were used for power analysis [80]. Based on the number of mediating variables for this study, as well as the correlation coefficients calculated between the four variables and the standard deviation coefficients for each variable, the sample size needed to achieve the 80% target power was calculated with a 95% confidence level. It was found that a sample size of approximately 120 was required. The sample size for this study was 328, which exceeds this recommended value, and therefore, the sample size can support the research hypothesis testing required. 

Then, the differences in the demographic characteristics of university students were compared according to gender, age, and learning level, respectively. It was found that in terms of emotional regulation, the mean value was significantly higher in males than in females, with a t-value of 4.280 (*p* < 0.01). In terms of psychological capital, males were significantly higher than females with a t-value of 2.009 (*p* < 0.05), and older university students were significantly higher than younger university students with an F-value of 4.196 (*p* < 0.05). In terms of learning satisfaction, there were no statistical differences between the different student groups. In terms of learning engagement, males were significantly higher than females with a t-value of 2.724 (*p* < 0.01), older university students were significantly higher than younger university students with an F-value of 8.033 (*p* < 0.01), and students with higher levels of study were significantly higher than students with lower levels of study with an F-value of 3.775 (*p* < 0.05). See Table 4.

**Figure 1:**
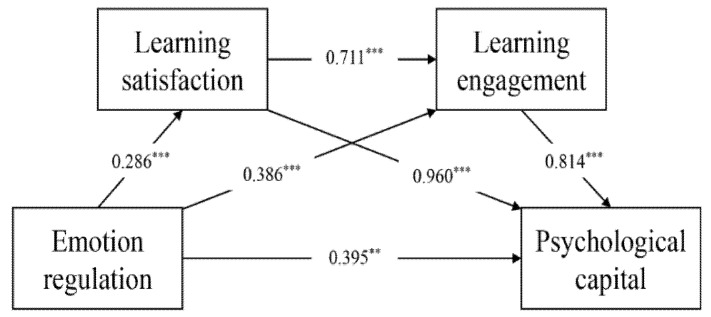
Mediation effect of learning satisfaction and learning engagement on the influence of emotion regulation on psychological capital. Note: ** *p* < 0.01, *** *p* < 0.001.

The data of the four variables were used for the regression analysis, and the results are shown in Table 5. Taking emotion regulation, learning satisfaction, and learning engagement as predictors, the regression variance of the psychological capital variable is significant (R^2^ = 0.576, F = 146.67, *p* < 0.001). Learning satisfaction and learning engagement positively predict psychological capital (B = 0.960, *p* < 0.001 and B = 0.814, *p* < 0.001, respectively; Figure 1).

The indirect mediation effect between emotion regulation and psychological capital is significant, and the indirect total effect is 0.754 (SE = 0.119, 95% CI [0.523, 0.998]). Further, the mediation effect of learning satisfaction as the mediating variable is significant, and the effect size is 0.275 (SE = 0.068, 95% CI [0.149, 0.421]). Meanwhile, the mediation effect of learning engagement as the mediating variable is significant, and the effect value is 0.314 (SE = 0.078, 95% CI [0.173, 0.479]). The mediation effect of learning satisfaction and learning engagement as mediating variables is significant, and the effect value is 0.165 (SE = 0.047, 95% CI [0.085, 0.268]). The ratio of the effect values of the three mediated paths relative to the total effect is 23.93%, 27.33%, and 14.36%, respectively. We found that the differences between the three indirect pathways are not significant (Table 6).

## 4. Discussion

The important role of psychological capital for the mental health of university students in the COVID-19 pandemic has been found by several studies [81,82]. However, less research has been conducted on the influence of the psychological aspects of learning on the relationship between emotion regulation and psychological capital in university students. This study explored the mediation effects of the learning satisfaction and learning engagement of university students on emotion regulation and psychological capital during the COVID-19 pandemic, which expands the research in this area [23,24]. We found that the learning satisfaction and learning engagement of university students play a mediating role in the impact of emotion regulation on psychological capital. 

Additionally, we found that there is a moderate correlation between the emotion regulation ability and psychological capital levels of university students, proving Hypothesis 1. This study demonstrated a correlation between emotion regulation and psychological capital among university students during the COVID-19 pandemic, supporting previous research [83,84]. The emotion regulation ability of people can promote mental health during the COVID-19 pandemic [85,86]. Further, reducing psychological distress and forming a more positive attitude is particularly important for the development and promotion of psychological capital [87]. From the conservation of resources theory perspective, people will consciously take measures to cope with challenges during a crisis. Maintaining positive and flexible emotions can avoid the loss of psychological resources and ensure recovery from setbacks more quickly. This may explain why the emotion regulation of university students can predict their psychological capital.

The findings of this study also show that learning satisfaction plays an intermediary role in the emotion regulation and psychological capital of university students, supporting Hypothesis 2. Previous studies have found correlations between emotion regulation and satisfaction [88,89] and between satisfaction and psychological capital [90,91], and these relationships were supported in the present study. Emotion regulation can help relieve stress, reduce negative emotions, and improve satisfaction. During the COVID-19 pandemic, academic pressure was an important factor that caused anxiety among university students [61]. The emotion regulation ability of university students may increase their satisfaction with learning, causing them to respond more actively to learning-related challenges, reduce the pressure from learning, and increase their self-confidence in learning. Further, from the personal resource theory perspective, stress has a negative impact on personal resources [92]. The learning satisfaction of university students can help them mitigate academic pressure and maintain positive psychological capital.

We found that the learning engagement of university students can play a mediating role in the impact of emotion regulation on psychological capital during the COVID-19 pandemic, supporting Hypothesis 3. The results show a positive correlation between emotion regulation and learning engagement, similar to previous studies [93]. Additionally, the emotion regulation ability of university students enables them to overcome negative emotions and maintain their learning state [94,95]. Emotion regulation ability may also assist university students in maintaining a positive attitude toward learning and enhance their enthusiasm for learning. From the resource retention theory perspective, the advantages for university students after dealing with emotional stress are conducive to pursing more learning achievements. A previous study conducted on work scenarios showed that work engagement may promote psychological capital [96]. Learning engagement can enable continuous learning achievements among university students, which may result in them being more optimistic and confident about the future, as well as help promote their psychological capital. This study found a correlation between student learning engagement and psychological capital, consistent with previous research findings [97,98]. Our research suggests that encouraging learning engagement among university students during the COVID-19 pandemic may be beneficial to their psychological capital.

We found that the learning satisfaction and learning engagement of university students play a continuous mediating role in the relationship between emotion regulation and psychological capital, supporting Hypothesis 4. We found that learning satisfaction was correlated with learning engagement, similar to previous studies [99,100]. The learning satisfaction and learning engagement of university students result in a positive learning state, which can promote student physical and mental development. Further, the good emotion regulation ability of university students also helps them maintain a positive learning state. A positive learning state helps students to perform better academically and may also assist them in obtaining better employment opportunities in the future, which makes them more optimistic and thus, promotes the continuous accumulation of psychological capital. During the COVID-19 pandemic, more learning support can promote the improvement of the psychological capital of university students. Specifically, disadvantaged students should be guaranteed learning opportunities [101], as a positive learning state plays an important role in mental health.

This research offers four main contributions. First, the study extends the theoretical understanding that emotion regulation predicts psychological capital by linking the emotion regulation of university students to psychological capital during the COVID-19 pandemic. Secondly, the study analyzed the mechanisms between emotion regulation and psychological capital during the COVID-19 pandemic and found that psychological aspects of learning, such as learning satisfaction and learning engagement, can play a sequential mediating role. In addition, this study explored important factors that predict the psychological capital of university students, which may provide a practical reference for universities to develop the psychological capital of students and promote their psychological well-being during the COVID-19 pandemic. Finally, this study also provides guidance for students to improve their psychological capital through learning, so that they can have feasible methods to cope with psychological challenges.

This research result provides useful practical enlightenment. Firstly, universities should help students master ways to regulate their emotions by offering mental health courses, popularizing emotion regulation methods, and providing psychological counseling to students. Secondly, universities should focus on enhancing the satisfaction of students with their studies, and more emphasis should be placed on improving educational resources during the COVID-19 pandemic to ensure that the studies of students are not affected. Teachers should optimize their teaching methods and focus on the needs of students for teaching and learning to increase student satisfaction with teaching. Finally, universities should help students plan their studies by optimizing their academic management and services and give them more academic support through teacher tutoring and peer support so that they can focus on their studies and ensure their learning engagement. Teachers should adjust academic tasks and assessment methods to reduce student academic stress, encourage students to face challenges positively, and improve their academic confidence. In conclusion, universities need to help students to have good mental health during the COVID-19 pandemic through both psychological interventions and academic support.

This study has some limitations. Firstly, it uses a cross-sectional study design, which makes it impossible to confirm the causal relationship between emotion regulation and psychological capital. Due to the methodological limitations, such mediating effects found in this study are not equivalent to causality, and longitudinal studies or experiments should be conducted in the future to examine this further. Secondly, this study aims to reveal an important mediating relationship between emotion regulation and psychological capital among university students, which also implies that there may be other potential influential paths between emotion regulation and psychological capital, and future studies should further analyze other potential mediating paths. Thirdly, this study may be biased because some university students who do not have the habit of using online social media platforms could not be collected, some busy university students may not be included in the study sample, and women are generally more involved in online research. There is a possibility that the findings cannot be generalized by the simple sampling method [102]. This makes it necessary to further validate the results of this study in the process of generalization to all university student populations in China or even other countries. In future studies, researchers can adopt stratified sampling to conduct large-scale surveys to reduce the sampling bias. Fourthly, students may not answer truthfully because of their unconscious social expectations or because they are vaguely aware of the purpose of the study through informed instructions, leading to research bias. For this reason, future studies should choose multiple data collection channels for corroboration. Fifthly, future studies should strengthen the analysis of the regulatory variables and explore the influence of the control variables. Finally, it is necessary to verify the conclusions of this study in other similar major global emergencies in the future.

## 5. Conclusions

This study used mediation effect models to analyze the mediation effect of the learning satisfaction and learning engagement of university students on emotion regulation and psychological capital during the COVID-19 pandemic. The results of this study found that the emotion regulation of university students can positively predict their psychological capital. Additionally, the learning satisfaction and learning engagement of university students can enhance the impact of emotion regulation on psychological capital. Further, the learning satisfaction and learning engagement of university students can form a correlation intermediary and have a mediation effect on the relationship between emotion regulation and psychological capital. This study shows that during the COVID-19 pandemic, in addition to short-term mental health education and intervention, attention should also be paid to improving education regulations, providing various support for the successful learning of students, promoting student learning satisfaction, improving learning engagement, and ultimately, improving their psychological capital and promoting their mental health.

## Figures and Tables

**Table 1:** The HTMT between emotion regulation, psychological capital, learning satisfaction, and learning engagement.

	Learning Engagement	Psychological Capital	Learning Satisfaction	Learning Engagement
Learning engagement	-			
Psychological capital	0.598	-		
Learning satisfaction	0.439	0.711	-	
Learning engagement	0.536	0.769	0.635	-

**Table 2:** Basic characteristics of participants.

Frequency	Category	Frequency	Percentage
Sex	Female	262	79.88%
	Male	66	20.12%
Age	18–22 years	273	83.23%
	23–25 years	48	14.63%
	26–37 years	7	2.13%
Learning level	Undergraduates	284	86.59%
	Master’s students	41	12.50%
	Doctoral students	3	0.91%
Location	Eastern area	139	42.38%
	Central area	102	31.10%
	Western area	73	22.26%
	Northeast area	14	4.27%

**Table 3:** The mean, standard deviation, range, and Pearson’s correlation of the study variables.

Variable	Mean of the Total Score	Mean Score of Each Item	Standard Deviation of Mean Item Score for Each Individual	Range of Mean Item Score for Each Individual	Emotion Regulation	Psychological Capital	Learning Satisfaction	Learning Engagement
Emotion regulation	44.79	4.48	0.62	2.50~6.30	1			
Psychological capital	122.34	4.71	0.7	2.50~6.69	0.395 **	1		
Learning satisfaction	45.33	3.78	0.56	1.75~5.00	0.268 **	0.629 **	1	
Learning engagement	56.32	3.31	0.58	1.59~4.76	0.375 **	0.684 **	0.548 **	1

Note: ** *p* < 0.01.

**Table 4:** Differences between student groups with different demographic characteristics in the total mean and standard deviation scores of emotion regulation, psychological capital, learning satisfaction, and learning engagement.

Variable	N	Emotion Regulation	Psychological Capital	Learning Satisfaction	Learning Engagement
Sex					
Female	262	44.06 ± 5.94	121.33 ± 17.80	45.23 ± 6.57	55.5 ± 9.26
Male	66	47.65 ± 6.62	126.33 ± 19.20	45.71 ± 7.09	59.59 ± 11.29
T		4.280 **	2.009 *	0.522	2.724 **
Age					
18–22 years	273	44.8 ± 6.09	121.72 ± 18.17	45.28 ± 6.54	55.95 ± 9.62
23–25 years	48	43.85 ± 6.50	123.04 ± 16.21	44.75 ± 7.00	56.31 ± 9.37
26–37 years	7	50.57 ± 8.02	141.57 ± 22.93	51.29 ± 7.25	70.71 ± 11.28
F		3.596 *	4.196 *	3.021	8.033 **
Learning level					
Undergraduates	284	44.76 ± 6.25	121.85 ± 18.32	45.29 ± 6.73	55.96 ± 9.78
Master students	41	44.39 ± 5.98	124.39 ± 16.58	45.1 ± 6.22	57.8 ± 9.59
Doctoral students	3	52.33 ± 6.35	140.33 ± 20.03	52.33 ± 3.79	70.33 ± 6.51
F		2.294	1.845	1.694	3.775 *

Note: ** *p* < 0.01, * *p* < 0.05.

**Table 5:** Regression analysis results.

Dependent Variable	Independent Variable	Unstandardized Regression Coefficients (B)	Standardized Regression Coefficients (β)	t	*p*	Bootstrap 95% Confidence Interval	R2	F	*p*
Learning satisfaction							0.072	25.24	*p* < 0.001
	Emotion regulation	0.286	0.268	5.024	*p* < 0.001	[0.164, 0.412]			
Learning engagement							0.356	89.819	*p* < 0.001
	Emotion regulation	0.386	0.245	5.311	*p* < 0.001	[0.231, 0.540]			
	Learning satisfaction	0.711	0.482	10.432	*p* < 0.001	[0.559, 0.857]			
Psychological capital							0.576	146.67	*p* < 0.001
	Emotion regulation	0.395	0.136	3.465	*p* < 0.001	[0.163, 0.616]			
	Learning satisfaction	0.960	0.352	8.112	*p* < 0.001	[0.688, 1.239]			
	Learning engagement	0.814	0.440	9.761	*p* < 0.001	[0.631, 1.002]			

**Table 6:** Test results of the mediation effect.

Pathway Type in the Model	Effect Size	BootSE	Bootstrap 95% Confidence Interval	Ratio of Effect Size to Total Effect
Direct pathway	0.395	0.114	[0.171, 0.619]	34.37%
Emotion regulation→Psychological capital				
Indirect pathways	0.754	0.119	[0.523, 0.998]	65.62%
Emotion regulation→Learning satisfaction→Psychological capital (ind1)	0.275	0.068	[0.149, 0.421]	23.93%
Emotion regulation→Learning engagement→Psychological capital (ind2)	0.314	0.078	[0.173, 0.479]	27.33%
Emotion regulation→Learning satisfaction→Learning engagement→Psychological capital (ind3)	0.165	0.047	[0.085, 0.268]	14.36%
Difference = ind1 − ind2	−0.04	0.116	[−0.274, 0.192]	/
Difference = ind1 − ind3	0.109	0.06	[−0.002, 0.234]	/
Difference = ind2 − ind3	0.149	0.088	[−0.023, 0.325]	/

Note: “/” represents no calculation.

## Data Availability

The data are not publicly available for privacy and confidentiality reasons. For reasonable data requirements, please contact the corresponding author.

## Supplementary Materials

The following supporting information can be downloaded at: https://www.mdpi.com/article/10.3390/ijerph192013661/s1, The Emotion Regulation Questionnaire [54], The University Students’ Learning Satisfaction Questionnaire [67], The Learning Engagement Questionnaire [75], Chinese Version of the Psychological Capital Questionnaire [60].
