# Supplementary material for: Emotion Regulation and Psychological Capital of Chinese University Students during the COVID-19 Pandemic: The Serial Mediation Effect of Learning Satisfaction and Learning Engagement
Source: Int J Environ Res Public Health. 2022 Oct 21;19(20):13661. doi: 10.3390/ijerph192013661 (PMC9603398; doi:10.3390/ijerph192013661)
Supplement: Supplementary file 1 [file ijerph-19-13661-s001.zip › ijerph-1914466-supplementary.pdf]

Supplementary Materials

The Emotion Regulation Questionnaire [54]

1.I control my emotions by changing the way I think about the situation I'm in.

2.When I want to feel less negative emotion, I change the way I'm thinking about the situation.

3.When I want to feel more positive emotion, I change the way I'm thinking about the situation.

4.When I want to feel more positive emotion (such as joy or amusement), I change what I'm thinking about.

5.When I want to feel less negative emotion (such as sadness or anger), I change what I'm thinking about.

6.When I'm faced with a stressful situation, I make myself think about it in a way that helps me stay calm.

7.I control my emotions by not expressing them.

8.When I am feeling negative emotions, I make sure not to express them.

9.I keep my emotions to myself.

10.When I am feeling positive emotions, I am careful not to express them.

The University Students' Learning Satisfaction Questionnaire [67]

1. I think what I have learned will be useful.
2. Most teachers are able to coordinate and control the teaching and learning process effectively.
3. I'm not satisfied with the school's teaching management system.
4. I have learned something useful at school.
5. I am satisfied with the teaching activities of most of the teachers.
6. I am satisfied with most of the teaching hardware facilities in the school.
7. The school's learning materials are useful to me.
8. The teaching style of most of the teachers meets my personal expectations.
9. The school has an appropriate timetable for lessons.
10. I am motivated to learn.
11. I can communicate easily with most teachers.
12. There are plenty of learning resources at the school.

Note: Item 3 is scored in reverse and the remaining 11 items are scored in forward.

The Learning Engagement Questionnaire [75]

1.When I get up in the morning, I feel like going to class.

2.When I’m doing my work as a student, I feel bursting with energy.

3.As far as my studies are concerned I always persevere, even when things do not go well.

4.I can continue studying for very long periods at a time.

5.I am very resilient, mentally, as far as my studies are concerned.

6.I feel strong and vigorous when I’m studying or going to class.

7.To me, my studies are challenging.

8.My study inspires me.

9.I am enthusiastic about my studies.

10.I am proud of my studies.

11.I find my studies full of meaning and purpose.

12.When I am studying, I forget everything else around me.

13.Time flies when I am studying.

14.I get carried away when I am studying.

15.It is difficult to detach myself from my studies.

16.I am immersed in my studies.

17.I feel happy when I am studying intensely.

Chinese Version of the Psychological Capital Questionnaire [60]

1. Many people appreciate my talents.

2. I don't like to be angry.

3. I have insights and abilities that exceed those of the average person.

4. I can recover quickly from setbacks.

5. I am confident in my abilities.

6. I rarely care about the unpleasantness in my life.

7. I can always accomplish tasks well.

8. Bad experiences can make me depressed for a long time.

9. When facing difficulties, I will calmly seek solutions.

10. I feel that I am tired of living.

11. I am willing to undertake difficult and challenging work.

12. When things don't go my way, I tend to get downhearted.

13. In times of adversity, I will actively try different strategies.

14. I can't eat well and sleep soundly when I'm under great pressure.

15. I am motivated to study or work to achieve my ambitions.

16. When the situation is uncertain, I always expect a good result.

17. I am working hard to achieve my goals.

18. I always see the bright side of things.

19. I pursue my goals with confidence.

20. I feel that good people are still the majority in society.

21. I have a certain plan for my studies and life.

22. Most of the time, I am high spirited.

23. I know exactly what kind of life I want.

24. I think life is good.

25. I don't know what my goals in life are.

26. I feel that the future is full of hope.

Note: Item 8,10,12,14,25 is scored in reverse and the remaining 21 items are scored in forward.

References [54,67,75,60] are cited in the Supplementary Materials
